# Does expanding fiscal space lead to improved funding of the health sector in developing countries?: lessons from Kenya, Lagos State (Nigeria) and South Africa

**DOI:** 10.1080/16549716.2018.1461338

**Published:** 2018-05-16

**Authors:** Jane Doherty, Doris Kirigia, Chijioke Okoli, Jane Chuma, N Ezumah, Hyacinth Ichoku, Kara Hanson, Diane McIntyre

**Affiliations:** a Health Economics Unit, University of Cape Town, Cape Town, South Africa; b School of Public Health, University of the Witwatersrand, Johannesburg, South Africa; c Kenya Medical Research Institute-Wellcome Trust Research Programme, Nairobi, Kenya; d Health Policy Research Group, Department of Pharmacology and Therapeutics, University of Nigeria, Nsukka, Nigeria; e Department of Economics, University of Nigeria, Nsukka, Nigeria; f Department of Global Health and Development, London School of Hygiene and Tropical Medicine, London, UK

**Keywords:** Fiscal space, tax collection, resource allocation

## Abstract

**Background**: The global focus on promoting Universal Health Coverage has drawn attention to the need to increase public domestic funding for health care in low- and middle-income countries.

**Objectives**: This article examines whether increased tax revenue in the three territories of Kenya, Lagos State (Nigeria) and South Africa was accompanied by improved resource allocation to their public health sectors, and explores the reasons underlying the observed trends.

**Methods**: Three case studies were conducted by different research teams using a common mixed methods approach. Quantitative data were extracted from official government financial reports and used to describe trends in general tax revenue, total government expenditure and government spending on the health sector and other sectors in the first decade of this century. Twenty-seven key informant interviews with officials in Ministries of Health and Finance were used to explore the contextual factors, actors and processes accounting for the observed trends. A thematic content analysis allowed this qualitative information to be compared and contrasted between territories.

**Findings**: Increased tax revenue led to absolute increases in public health spending in all three territories, but not necessarily in real per capita terms. However, in each of the territories, the percentage of the government budget allocated to health declined for much of the period under review. Factors contributing to this trend include: inter-sectoral competition in priority setting; the extent of fiscal federalism; the Ministry of Finance’s perception of the health sector’s absorptive capacity; weak investment cases made by the Ministry of Health; and weak parliamentary and civil society involvement.

**Conclusion**: Despite dramatic improvements in tax revenue collection, fiscal space for health in the three territories did not improve. Ministries of Health must strengthen their ability to motivate for larger allocations from government revenue through demonstrating improved performance and the relative benefits of health investments.

## Background

As international support for Universal Health Coverage (UHC) grows apace, the issue of how to finance improved financial protection and access to needed health services becomes ever more urgent [1,2]. There is growing recognition that low- and middle-income countries must find additional public domestic sources of finance if they want to make tangible and sustained progress towards UHC, given the relatively low levels of public funding for health care in many of these countries at present and the challenges arising from a heavy dependence on donor funding [3–5]. Thus, there is an increased focus on how to create fiscal space for health, which ‘refers to the capacity of government to provide additional resources for health without jeopardising its long-term financial position and economic stability’ [6,7]. For this article, the review undertaken for the research project up to 2012 [4] was supplemented by a further review for the period 2013–2017. A search using the terms ‘fiscal space’ AND ‘health’ was conducted on EBSCOHost (Academic Search Premier, CINAHL, EconLit and Medline) and PubMed. Articles included for full review were those that focused on mechanisms for increasing government spending on health care from domestic sources. Articles focusing on the allocation of resources within the health sector, between different health services or geographic areas, were excluded as were articles focusing on funding for specific services (e.g. HIV services and immunizations) and those not in English. The literature on fiscal space for health has focused on improving the conceptual understanding of fiscal space, providing guidelines for how to assess the fiscal space for health in individual countries, and cross-country comparative analyses of the health sector’s share of government expenditure [4]. From a conceptual perspective, other than achieving efficiency gains within the health sector [7,8], fiscal space for increased domestic funding of health services requires increases in overall government revenue and expenditure and/or an increasing share of government resources being devoted to the health sector (i.e. greater prioritisation of the health sector by government) [4]. Economic growth contributes to increases in government revenue and expenditure, unless tax rates are reduced, and to increases in government funding of health services if the percentage share of government spending on health remains constant or increases [4,9]. An econometric analysis of different Indian states found that per capita GDP growth was the most important determinant of health spending increases [9]. However, while economic growth is critical for improved fiscal space and while countries with higher GDP per capita levels tend to have higher levels of government expenditure, there is considerable variation in government revenue and expenditure as a percentage of GDP between countries with similar income levels [10]. This points to the importance of fiscal policy and government choices about what taxes to levy and at what rate [10].

There is a growing literature exploring ways in which government revenue could be increased, ranging from improving tax revenue collection and administration efficiency, to increasing rates of existing taxes and introducing new taxes or other government revenue sources, such as from the extraction of natural resources [3,10–20]. Some studies model the additional government revenue that could be generated through such strategies, and the potential increases in government health expenditure [19,21,22]. These studies demonstrate that it is possible to improve fiscal space for health services. For example, one study has estimated that if tobacco taxes were increased, government spending on health care could increase by 4% globally [8].

However, a key assumption in these models is the prioritisation given to health relative to other sectors in government’s resource allocation decision-making [21]. Even if government revenue and overall government expenditure increases, this does not automatically translate into increased public spending on health services. This is entirely dependent on government decisions, particularly by the Ministry of Finance, about the relative priority of the health sector.

The literature on the factors influencing the prioritisation of health in government budgets is surprisingly scant, with our literature search identifying very few studies published in the peer-reviewed literature on these factors in low- and middle-income countries. This is a striking gap, given that most African countries fall far below the 15% target set by the Abuja Declaration of 2001 [23], and that considerable attention is being paid to improving the tax collection capacity of low- and middle-income countries [13–15]. This begs the questions: which sectors are benefiting from improved fiscal space, what explains the public health sector’s position in this competition for more resources, and how can Ministries of Health influence resource allocation decision-making in their favour?

This article makes a contribution to knowledge in these areas. It is based on a collaborative research project undertaken by the RESYST (Resilient and Responsive Health Systems) research consortium in Kenya, Lagos State (Nigeria) and South Africa (More information on RESYST can be accessed at http://resyst.lshtm.ac.uk).

All three territories had successfully implemented tax generation and collection reforms over the past two decades, following dramatic changes in their political systems (see Box 1 for a summary of health financing mechanisms and tax collection reforms in these territories).

This article examines whether improved government revenue in these three territories was mirrored by increased resource allocation to their public health sectors, and if not, which sectors did benefit from increased allocations, including donor funds flowing through government channels. It also explores the factors influencing decisions on the allocation of government funds to the health sector. It provides recommendations for Ministries of Health to strengthen budget allocations to the health sector, which are also likely to be of interest to Ministries of Finance.

## Methods

The case study territories were chosen primarily because of their track record in improving government revenue generation, but also because, during the period under review, they represented low-income (Kenya), lower middle-income (Nigeria) and upper middle-income (South Africa) settings as well as diverse regional settings, namely West, Eastern and Southern Africa, respectively. They also represented different levels of political decentralization: at the time it was studied, Kenya still had a centralized political and health system (although this has since changed with the devolution of authority to county level in 2013), whereas Nigeria and South Africa have quasi-federal systems where the health function is shared between different levels of government (the difference being that Lagos State, unlike the provinces of South Africa, is able to generate substantial revenue internally). It must be noted that, since this research was undertaken, Kenya has been reclassified as a middle-income country by the World Bank (in 2014).

A common mixed methods approach was developed by the research team to ensure that a comparable set of quantitative and qualitative data was compiled and consistent analysis undertaken in each case study territory. For example, a standardised data extraction template was used across the three case study territories for documenting the quantitative data on trends in government revenue, total expenditure and sectoral expenditure. Similarly, a semi-structured interview schedule was used for qualitative data collection on the factors influencing allocations to the health sector.

For this article the review undertaken for the research project up to 2012 [4] was supplemented by a further review for the period 2013–2017. A search using the terms ‘fiscal space’ AND ‘health’ was conducted on EBSCOHost (Academic Search Premier, CINAHL, EconLit and Medline) and PubMed. Articles included for full review were those that focused on mechanisms for increasing government spending on health care from domestic sources. Articles focusing on the allocation of resources within the health sector, between different health services or geographic areas, were excluded as were articles focusing on funding for specific services (e.g. HIV services and immunizations) and those not in English.

### Quantitative data collection and analysis

Quantitative data were extracted from each territory’s official government revenue and expenditure reports, such as the Accountant-General or Auditor-General reports. Extracted data included the following variables: total government revenue, total government expenditure (using actual rather than budgeted expenditure), government expenditure on health, and government expenditure on other key sectors such as education, social protection and justice, and security services. While each territory uses slightly different terminology for specific health activities, the research team carefully compared health services included in reports from each country to achieve the greatest comparability possible.

Data on these indicators were extracted for at least a 10-year period in each territory to describe trends. The exact time-period selected depended partly on the availability of data in each territory and the period of tax reforms, but was generally from the mid-1990s to the end of the first decade of this century. All three case studies included at least the years 2000 to 2010. Various analyses were undertaken on these variables, including: calculating annual percentage changes and assessing trends in real total revenue and expenditure and real per capita expenditure, where the term 'real' refers to removing the effect of inflation. All real data are presented in 2010 terms.

**Figure 1.**
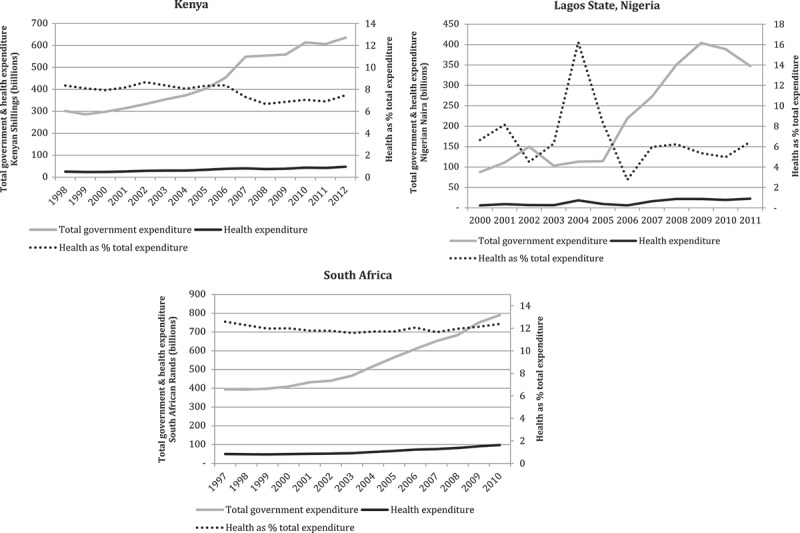
Changes in the proportion of total government expenditure allocated to the health sectors of Kenya, Lagos State and South Africa (2010 prices).

It should be noted that with respect to Lagos State, it was not possible to determine all sources of health financing received (including federal funds), because national data do not allow this level of disaggregation. The Lagos State analyses are therefore confined to internally-generated revenue, which, in the case of the health sector, is primarily spent on hospital services. This limits opportunities for comparison with Kenya and South Africa, where the analyses capture spending on the entire health system, including from national, regional and local sources. Further, during the period under review Lagos State periodically spent a very large portion of its total budget on capital projects, which distorted comparative analysis of health spending as a percentage of total government budgets across the three territories. Nonetheless, some quantitative data from Lagos State are presented where they are pertinent (for example, in Figure 1), and in the online supplementary data file, so that they are available to other researchers seeking to obtain greater clarity on spending patterns in this under-researched geographic area.

### Qualitative data collection and analysis

The qualitative component of the case studies entailed 27 key informant interviews across the three territories with purposively selected officials in the Ministries of Health and Finance with knowledge of government resource allocation processes. Semi-structured interview guides were used, with questions focused on the processes and actors involved in decision-making on the allocation of government funds to the health and other sectors, and the political, economic and other contextual factors influencing these decisions. Interviews were audio-recorded, transcribed and subjected to thematic content analyses that drew on the key components of the policy analysis framework of Walt and Gilson [24]. This framework organises the various influences on the impact of a policy according to context, the design of the policy, the power, interests and behaviour of various actors or stakeholders, and the process of policy development and implementation. The framework also encourages an appreciation of the interaction between these factors.

### Ethical issues

Ethics approval was obtained from the relevant academic institutions in each of the case study territories, and from the London School of Hygiene and Tropical Medicine, the coordinating institution for the study. Informed consent was obtained from key informants.

## Results

Given the mixed methods approach adopted in this study, in this section the results from the quantitative component of the study are presented in an integrated way with the results from the qualitative component. The qualitative findings explain the trends in government revenue and spending in terms of the most important factors relating to the context, actors, policy design and process, and the interaction between them (Walt and Gilson [24]).

### Trends in government revenue and spending on the public health sector

Government revenue generation increased dramatically in each of the three territories in the first decade of the 21st century (see Table 1): the reasons for this are detailed elsewhere [16-18]. By 2010 Kenya had experienced a 57% increase in revenue in real terms, compared to 2000, as the result of an average annual percentage increase of 5%. The equivalent figures for South Africa were 71% and 6%, respectively, while revenue in Lagos State increased by a dramatic 207% as a result of an average annual percentage increase of 13%.

**Table 1.** Changes in revenue collection by territory in real terms (2010 prices), 2000–2010.

Indicator	Kenya	Lagos State (Nigeria)	South Africa
Average annual percentage change	4.7	12.7	5.7
Change over entire period, as a percentage	57.3	206.8	71.4

Analysis of data presented in Doherty (2015) [16], Kirigia, Munge and Chuma (unpublished) [18] and Lagos State Accountant-General’s Reports. Analysis of data presented in [16–18].

As shown in Figure 1, total government expenditure and absolute levels of government health care expenditure increased in real terms in all three territories over the periods of revenue increases.

However, Figure 1 also indicates that in all territories health care expenditure increased much more slowly than general government expenditure. Thus, in Kenya, Lagos State and South Africa, the health sector was not able to increase its percentage share of the total government budget over the study period. Instead, the health sectors of all three territories lost some ground at certain points. In Kenya, the health sector’s highest percentage share of total government expenditure was 8.4% (in 2006), while its lowest was 6.7% (in 2008). The health sector’s proportion in South Africa was considerably higher, at a maximum of 12.6% (in 1996), but reached a low point of 11.6% (in 2003). In Lagos State, where the percentage share of total government expenditure was much more volatile, the health share of expenditure peaked at 8.4% in 2005 but was as low as 2.3% in 2009. As already mentioned, the volatility of the Lagos State percentages is partly an artefact of the large capital projects undertaken by the Lagos State government in some years. For this reason, the rest of the quantitative analyses presented in this article exclude data from Lagos State (these data are presented in an online supplementary file for the sake of completeness).

**Figure 2.**
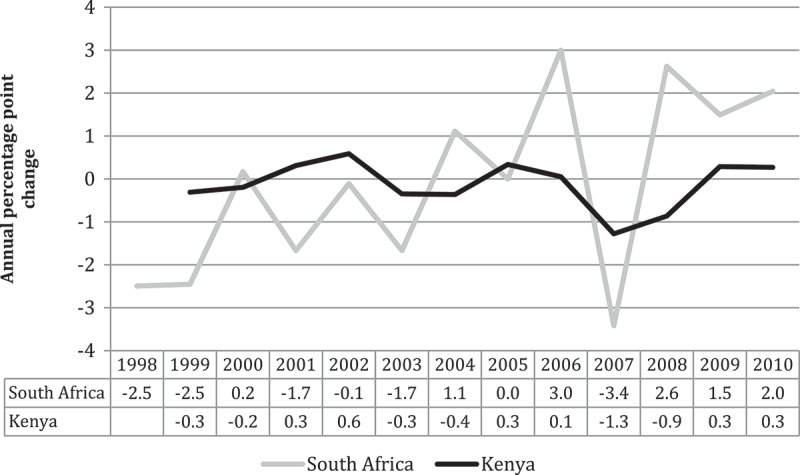
Annual percentage point change in the percentage of total government expenditure allocated to the health sectors of Kenya and South Africa (2010 prices). Adapted from [16] and [18].

Figure 2 underlines the limited fiscal space enjoyed by the Kenyan and South African health sectors by showing the annual percentage change in the relative share of each country’s health budget. Kenya’s share remained relatively stable (as the annual percentage change was close to zero throughout the period), while the South African public health sector experienced slightly more variation year on year (suffering from negative growth in earlier years, but improving its share slightly towards the end of the study period).

**Figure 3.**
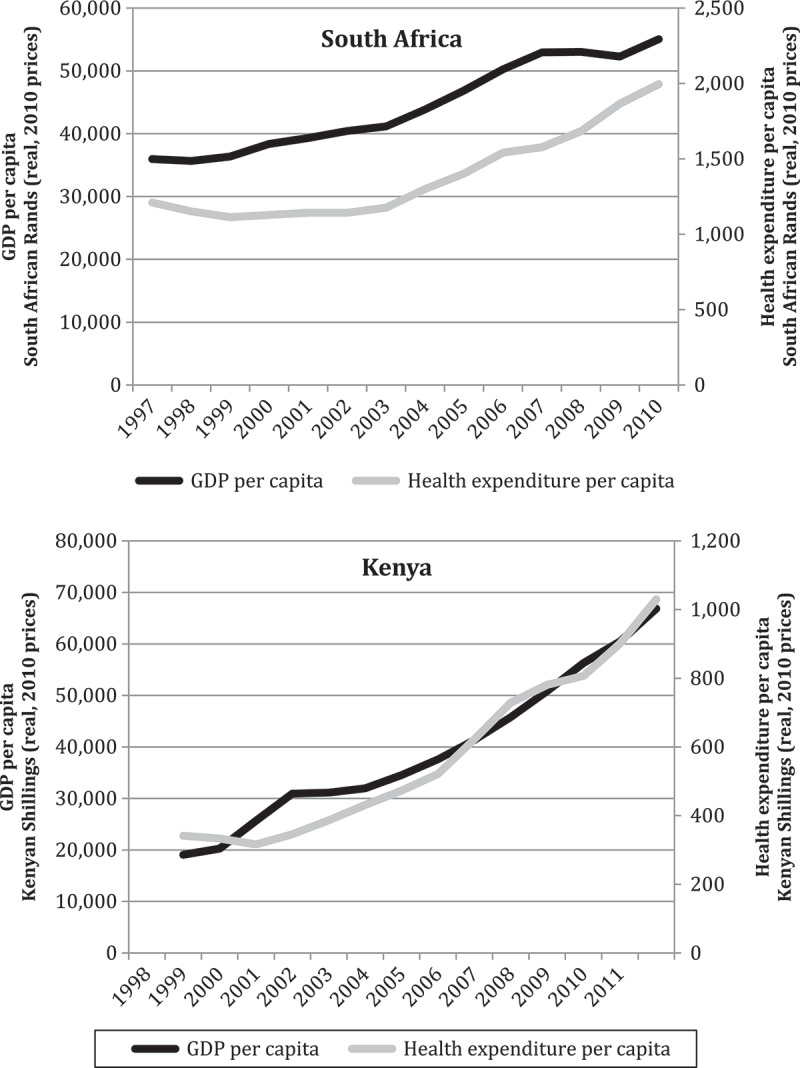
Comparison of the trend in real GDP per capita and health expenditure per capita in Kenya and South Africa (2010 prices). Adapted from [16] and [18].

In South Africa, real per capita government spending on health actually declined over part of the study period, followed by a recovery (see Figure 3). Thus, per capita health spending fell from R1210 in 1997 to a low of R1113 in 1999, only surpassing the 1997 level again in 2004 (real terms, 2010 prices). This was in the context of expanding per capita GDP over the same years, except in 1998. While the Kenyan data show an early decline in Figure 3, the growth in per capita health expenditure begins to mirror the growth in GDP per capita much more quickly than in South Africa.

**Figure 4.**
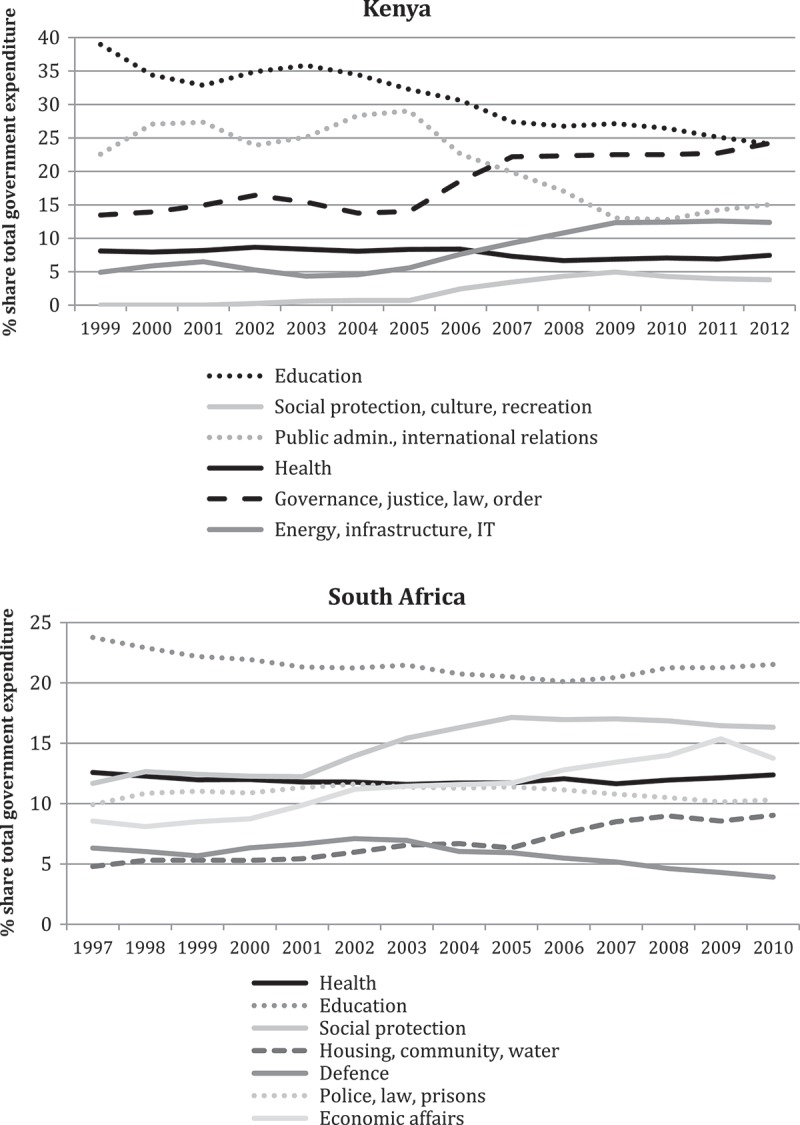
Percentage shares of the total government budget for key sectors in Kenya and South Africa (2010 prices). Adapted from [16] and [18].

Figure 4 shows the relative allocations of government expenditure between selected key sectors in each of Kenya and South Africa. Unfortunately strict comparison is not possible. This is because governments classify their different sectors differently. However, the intention is to show the relative priority given to the health sector in each country, rather then to compare the different non-health sectors between countries.

In Kenya, health spending was much lower than education spending, although the latter declined rapidly over the study period. On the other hand, spending on the governance and public safety-related portfolios expanded rapidly. Spending on social protection, energy and infrastructure also grew. The health sector remained a relatively low priority over the study period, starting off in fourth position and ending up in fifth position.

The situation in South Africa also saw the public health sector falling in the list of priorities by the end of the period (where it was fourth out of the selected sectors), compared to the beginning (where it was second). Social protection and economic affairs were the sectors that usurped the health sector’s position, but rapid growth in the local-government related sectors of housing and water were also a feature of this period.

The above analyses show that while government revenue and total government expenditure increased substantially over the study period, the health sector did not benefit from these increases as much as many other sectors. This indicates that the relative priority accorded to the health sector by all three governments declined over the study period and that the fiscal space for health consequently shrank in all three territories. It is particularly concerning that real per capita government health spending declined in South Africa, for 6 years in the period under review, despite overall increases in tax revenue, even as the country witnessed the explosion of the HIV/AIDS epidemic. The qualitative data presented in the next section explore why the health sector in the three case study territories received a relatively low priority in government resource allocation decisions.

### Factors that affected allocations to the public health sector

In all three territories, a new spirit of political openness and accountability linked to democratic changes provided favourable circumstances for increased revenue collection and opportunities for health system reform [16–18,25]. Key informants attested that all three governments acknowledged the severe health challenges they faced, the failures of their current health system and the political importance of health (Box 2).

Why then were the public health sectors of these three territories not able to attract a greater share of resources, let alone retain their share of government funding? The answer seems to lie in the complex interplay between economic, political and administrative issues that determine budgetary allocations to various sectors, what one could call ‘the political economy of the budget allocation process’.

First, government development policies influenced allocations to the health sector. In Lagos State, this took the form not only of competition between sectors but also of massive expenditure on governance, administration and, above all, infrastructure, that crowded out spending on the selected social sectors. A Nigerian key informant explained that,

‘it’s the government that has a vision of what we want to do….I mean the vision of eliminating … reducing poverty using infrastructural development as a tool for poverty alleviation’.

In South Africa, by way of contrast, the early years saw macro-economic policy cap the tax to GDP ratio, constrain overall government expenditure, require rapid servicing of government debt (which squeezed out sectoral spending) and elevate the role of business and infrastructure in achieving economic growth [26]. Interviewees from both these territories emphasized, however, that spending in several sectors outside the health sector (such as housing, water and sanitation) could be interpreted as having contributed to health by addressing the social determinants of ill-health.

A second factor that affected resource allocation was the fiscal federal nature of Lagos State and South Africa. For the former, interview data indicated that the process of creating a new state government seems to have both consumed enormous resources and made tracking sectoral spending difficult, given different functions and accounting systems at the federal and state levels. In South Africa, fiscal federalism, which was not as complete as Lagos State’s separation of powers and responsibilities, nonetheless introduced a number of technical and political barriers to protecting the health sector’s share of revenue across the different levels of government. In particular, each provincial government has authority to determine its own allocations across sectors and provinces assign different priorities to the health sector relative to other sectors which impacts on total government health spending levels [27]. Kenya had a centralised political and health system during the period under review, but fared no better in protecting resources for health, suggesting that national budget processes – with their iterative cycles of technical analysis and political bargaining – are difficult to understand and influence in favour of health spending in any setting.

A third factor affecting resource allocation decisions in South Africa was clearly the personal attributes of the different Ministers of Health. Some Ministers were politically well connected and able to gain the trust and support of their Cabinet colleagues, whereas other Ministers were not nearly as effective. As one South African interviewee put it:

‘to be a successful Minister, yes, you must have your own vision and you must be passionate about that vision, but actually a critical point is to turn what’s your pre-occupation, your vision, into a shared collective vision of your Cabinet colleagues’.

Fourthly, the Ministry of Finance’s perspective on national priorities and the performance of the Ministry of Health was a crucial factor in all three territories. A Nigerian interviewee described the frustration felt by the Ministry of Finance when receiving motivations from the Ministry of Health (MOH) for additional resources, when some existing funds remained unspent and had to be re-allocated to other needy sectors:

‘the MOH has weak absorptive capacity so even with the allocated budget you will find that MOH is one of the sectors that will return funding due to underutilization…this could be due to internal politics within the MOH or delayed projects approvals…this is the Ministry that has many challenges when it comes to timely budget utilisation…but they always argue that funds given to health are insufficient …’

Inefficiencies are not always the fault of the Ministry of Health, however: delays in the disbursements of funds by the Ministry of Finance, especially towards the end of the financial year, can also be a factor, as noted by both Kenyan and Nigerian interviewees.

Fifthly, this study found that Ministries of Health were seldom able to mount strong technical arguments in support of their bids for more resources. In both Kenya and South Africa, the technical experts who were involved in budget formulation were not involved in budget presentations at which the Ministry had to defend its proposals. There were also few health economists in either Ministry, which added to the problem of inexperienced people preparing budgets with weak justifications, especially with the shortage of adequate data on health needs and outcomes. As a South African key informant explained:

‘There’s a very nice complex interplay between your commitment as a Minister and the strength of your technical argument when you try to convince your colleagues that this is the right direction to go … No matter how right or passionate one individual Minister might be … Ministers get lobbied, Ministers are looking after their own portfolio in the short term, and so you need to be able to pursue them and show them … the strength of that argument’.

In any case, demonstrating the impact of health spending is easier said than done, as one Kenyan official described:

‘… [an] increasing health budget is important but will the higher funding translate into better performance in the health sector? What we need is to demonstrate that resources allocated to health are distributed efficiently to achieve the desired health outcomes and particularly reducing health inequalities among poor and vulnerable people… Unfortunately the current budgeting system does not include a mechanism for tracking health expenditure to ensure it responds to the needs of the poor which is important for accountability and equity…’

Finally, none of the territories benefited from strong and coordinated parliamentary and civil society campaigns for the realisation of access to health care (a notable exception being the campaigns for access to HIV/AIDS treatment in South Africa). Labour unions and professional associations did mobilise for improved health worker salaries in Lagos Sate and South Africa, which in some ways helped to protect the health budget but in others compromised the funding available for other aspects of service delivery [16,17].

## Discussion

The Kenyan, Lagos Sate and South African experiences show that, with transformation of their tax collection agencies and related reforms, it was possible to increase total revenue collection quite dramatically. This created a fiscal climate where it became possible to countenance increased government expenditure on priority programs. However, none of the three territories’ public health sectors was able to increase its relative share of government resources, and each saw its share decline over several years of the study period. This was despite the considerable health and health care problems faced by each country, and relatively low levels of government health expenditure in international terms.

None of these territories managed to reach the 15% target set by the Abuja Declaration of 2001, although South Africa, as an upper middle-income country, came the closest. While there is some, albeit limited, literature on the lack of progress towards the Abuja target in most African countries [28,29], there is a dearth of empirical research on why the health sector appears to face more severe fiscal space constraints than many other sectors.

A key contribution of this article is that it explores why this was the case in three African territories, even though addressing the failings of their health systems was a priority for each of the territories’ governments, through interviews with key informants in the Ministries of Health and Finance. Factors that played a part included the context of fiscal policies that favoured growth in so-called ‘productive’ sectors, the extent of fiscal federalism, Ministries of Health that had insufficient capacity to demonstrate the health sector’s positive contribution to development, Ministries of Finance that were distrustful of the absorptive capacity of the public health sector and its ability to deliver, and weak parliamentary and civil society involvement.

These findings resonate with a study conducted in India, which also found that fiscal federalism, with autonomy at State level in inter-sectoral allocations, adversely affected fiscal space for health, and that direct allocations from the national level for priority health programs were helpful in increasing overall government spending on health services [30]. This study also identified inadequate absorptive capacity in the health sector as contributing to lack of growth in government spending on this sector.

The only other literature commenting on these issues in the African context that could be located is a paper based on a series of panel discussions between Ministries of Finance and Health [31]. The key message from these panel discussions was the need for Ministries of Health to build an evidence base on the need for investment in the health sector and to demonstrate efficient use of resources within the health sector. This supports the findings presented from this research.

A key limitation of the research reported in this paper is the inability to obtain comprehensive data on public health expenditure in Lagos State. This only affected the quantitative component of the findings. Another limitation of the study overall is that it was difficult to access some of the most senior government officials for interviews because of their heavy workloads.

## Conclusions and policy implications

This research highlights that increases in tax revenue collection do not necessarily translate into improved fiscal space for health. The onus is firmly on Ministries of Health to address this by demonstrating good performance and making an investment case for health.

To strengthen their bargaining position in the budget-setting process, Ministries of Health need to develop their capacity to mount and defend bids for improved funding. This involves improved technical capacity (especially in health economics) and the timely involvement of technical experts in budget negotiations. It also involves clear and persuasive interventions by the Minister of Health in Cabinet policy-making processes.

The Ministry of Health needs to address concerns, especially from the Ministry of Finance, that the public health sector is unable to spend funding it receives, or spends it inefficiently, by documenting successes in service delivery. It also needs to counter these arguments by demonstrating the debilitating effects of underfunding on service capacity.

Ministries of Health, with the support of civil society, also need to develop clear advocacy strategies for the public health sector. Importantly, they need to engage with debates on appropriate macro-economic choices. The trade-offs between developing public health services and growing the economy must be made explicit. Ministries of Health need to convince Cabinet and the Ministry of Finance that investing in health is a productive choice that helps to grow the economy and achieve development goals.

## 

**Box 1.** Overview of health financing and recent tax reforms in case study territories.

Overview of health financing
The three case study countries have a very different distribution of health financing across funding sources. Although data are not available for Lagos State, data for Nigeria as a whole provide insights into the likely health financing context in Lagos.
According to the WHO’s National Health Accounts database, health services in Nigeria are largely funded through direct out-of-pocket payments by individuals to health care providers, which accounts for 72% of total health expenditure (THE). Mandatory pre-payment funds (tax revenue allocated to health and sometimes mandatory health insurance contributions) account for 25% of THE, with private health insurance and other private funding (e.g. of non-governmental organisations) only accounting for 3% of THE.
Kenya has the highest share of mandatory prepayment funding (61% of THE) of all three countries, with out-of-pocket payments accounting for 26% of THE and private health insurance and other private funding for 13% of THE.
In South Africa, there is an almost equal distribution of health care financing between mandatory prepayment funding (48% of THE) and private health insurance (45% of THE), with the lowest share of out-of-pocket payments (7% of THE) of all three countries.
Unfortunately the WHO database does not distinguish what component of mandatory prepayment funding is from domestic (tax or mandatory health insurance) sources or from external donor funding channeled through government. Donor funding may also flow to non-governmental organisations. However, it is known that South Africa and Nigeria have very limited donor funding (2% and 7% of THE, respectively) while Kenya is quite dependent on donor funding (28% of THE).
Overview of tax reforms
A core component of the reforms in all three territories was organisational reform of the respective tax collection agencies, particularly granting semi-autonomy to these agencies, improving their capacity including through improved IT systems, and striving to achieve an organisational culture of zero tolerance for corruption [16-18]. Multiple strategies to promote tax compliance were also introduced, and tax enforcement was strengthened. All three territories expanded their tax base, with Kenya and Nigeria introducing tax collection from the informal sector.
.

**Box 2.** Political support for the health sector in all three territories.

‘In a critical policy document known as the Kenya Vision 2030 launched in 2008, the government of Kenya committed to improve the overall livelihoods of Kenyans by providing an efficient and high quality health care system with the best standards’ (Kenyan key informant)
‘The governor is very sensitive to health [so] that he had to increase the fund to be focused to the health sector, because health is wealth, and they are very particular of the health of the people of Lagos state.’ (Key informant from Lagos State)
‘There’s no question that during the Madiba [Mandela] presidency and also moving into the Mbeki presidency, there was a great recognition of the role that health could play … Madiba also looked at it from the perspective that it was also one of those sectors where you could achieve much within a generation and therefore was critical to the process of nation-building and the restoration of people’s dignity’ (South African key informant)

## Paper context

African countries require additional domestic sources to provide universal health coverage. This paper wanted to find out whether increased tax revenue collections in three African territories benefited the health sector. It was surprising to find that the health sector’s share of government budgets declined over the period under study. The authors explored why this was so, generating recommendations for Ministries of Health seeking to grow the health sector’s share of available government funds.
